# Immobilization of molecular catalysts for artificial photosynthesis

**DOI:** 10.1186/s40580-020-00248-1

**Published:** 2020-11-30

**Authors:** Dong Ryeol Whang

**Affiliations:** Department of Advanced Materials, Hannam University, 34054 Daejeon, Republic of Korea

**Keywords:** Artificial photosynthesis, Molecular catalysts, Immobilization, Solar fuel

## Abstract

Artificial photosynthesis offers a way of producing fuels or high-value chemicals using a limitless energy source of sunlight and abundant resources such as water, CO_2_, and/or O_2_. Inspired by the strategies in natural photosynthesis, researchers have developed a number of homogeneous molecular systems for photocatalytic, photoelectrocatalytic, and electrocatalytic artificial photosynthesis. However, their photochemical instability in homogeneous solution are hurdles for scaled application in real life. Immobilization of molecular catalysts in solid supports support provides a fine blueprint to tackle this issue. This review highlights the recent developments in (i) techniques for immobilizing molecular catalysts in solid supports and (ii) catalytic water splitting, CO_2_ reduction, and O_2_ reduction with the support-immobilized molecular catalysts. Remaining challenges for molecular catalyst-based devices for artificial photosynthesis are discussed in the end of this review.

## Introduction

Natural photosynthesis provides over 99% of nutrients to the living organisms on earth. At the beginning of food chains, photosynthetic autotrophs, i.e. plants, algae, and bacteria, convert inorganic substances into energy-intensive food by using sunlight as an energy source. Through the course of 3.4 billion years [1] nature has selected molecular systems to carry out the photochemical processes. Molecular catalysts have abundant advantages: (i) well defined molecular structure and active center (ii) feasible mechanistic study with the help of various spectroscopic techniques, (iii) maximized active sites in homogeneous solutions, (iv) high tunability of their chemical/physical properties by rational molecular design, etc. [2]. These features inspired the development of molecular catalysts for solar energy transduction in artificial photosynthesis.

The concept of storing the solar energy into higher-energy chemicals is the basis of artificial photosynthesis, which is dedicated to producing fuels/high-value chemicals such as H_2_, methane, methanol, or hydrogen peroxide. A number of molecular catalysts have been developed for water splitting [3–12], CO_2_ reduction [13–21], and O_2_ reduction [22, 23]. However, the molecular catalysts in homogeneous solution often suffer from low stability in redox conditions. In addition, debates have been made whether the organometallic catalysts are true homogeneous catalysts or just a precursor of heterogeneous catalysis [24]. One important implication of natural photosynthetic system is that the molecular cofactors are optimally positioned in a protein matrix, so the photochemical processes are carried out in an efficient and robust way [25–28]. Although replicating the complicated configuration of natural photosystem is not that straightforward, more simple chemistry was addressed to immobilize molecular catalysts in solid matrices, i.e., covalent bonds and non-covalent interactions. This review revisits the techniques for immobilizing molecules in solid supports and summarizes recent advances in artificial photosynthesis using the techniques.

## Immobilization methods

### Covalent immobilization

**Scheme 1:**
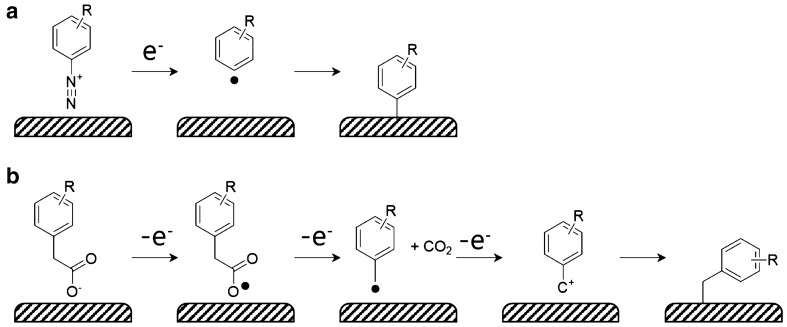
Covalent attachment of **a** aryl diazonium salts and **b** aryl acetates onto carbon surface

Covalent bond between a catalyst and its support can result in stable immobilization. Several synthetic methods have been proposed for covalent attachment of molecular catalysts onto carbon-based surfaces [29–32]. A widely used strategy to for direct C–C bond formation was pioneered by Pinson and Savéant is based on electrochemical grafting based on reduction of aryl diazonium molecules. The diazonium salts are reduced on the surface of carbon-based electrode to form aryl radicals, which reacts with the carbon surface to form stable C–C bonds [31–33]. This method was successfully adopted for the preparation of quinone decorated electrodes showing electrocatalytic activities for catalytic oxygen reduction (Scheme 1a) [34–39].

An alternative approach based on electrochemical oxidation of aryl acetates was reported [40–42]. The detailed mechanism is more complicated compared to the diazonium pathway and still under debate [42, 43]. González group proposed one possible mechanism, where anodic oxidation of carboxylates results in removal of CO_2_, thus yields arylmethyl radical. The radical can be further oxidized to form methyl carbocation, which forms C–C bond with carbon surface (Scheme 1b) [42].

**Fig. 1:**
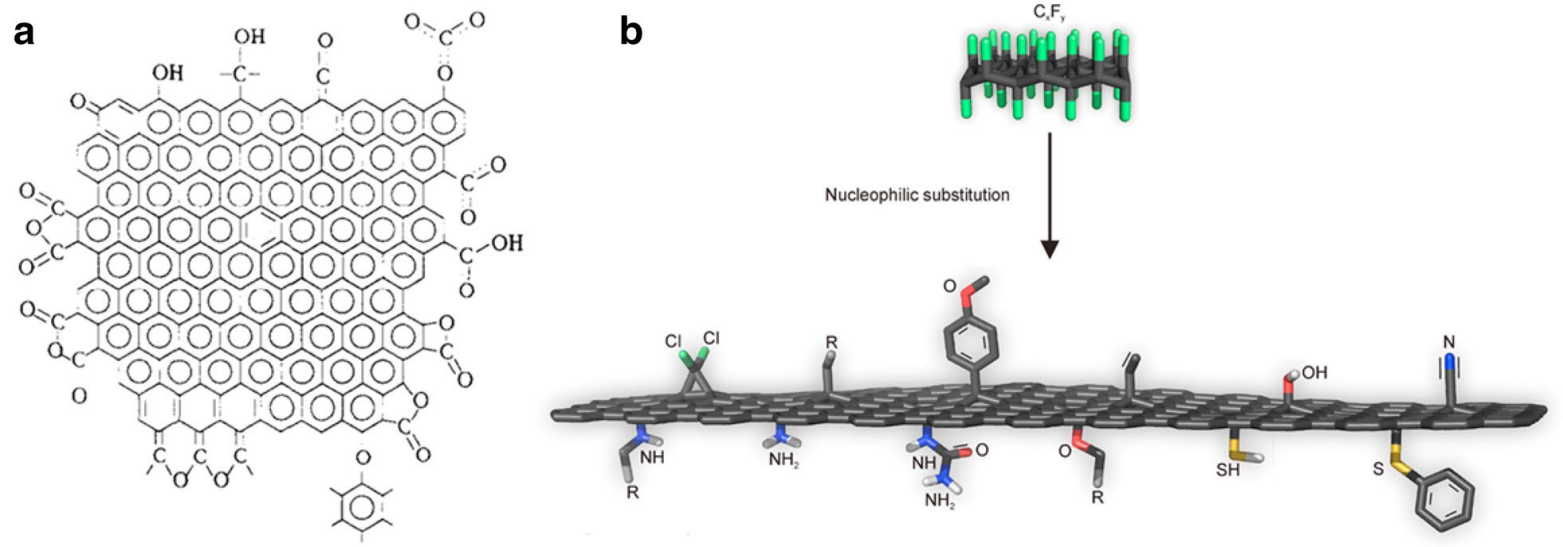
**a** Oxidative functionalization of carbon surfaces. Reproduced from [44] with copyright permission from 1993 Elsevier Ltd. **b** Preparation of graphene derivatives from fluorographene. Reproduced from [54] with copyright permission from 2017 Elsevier Ltd.

Pre-functionalization of carbon materials provides synthetic feasibility to incorporate molecular catalysts. Oxidation of carbon surface provides oxygen-containing groups, such as ketone, quinone, phenol, lactone, carboxyl group, ether, acid anhydride, ketene, etc. [44], which can serve as anchoring sites for subsequent functionalization with molecular catalysts (Fig. 1a). More recently, fluorinated graphene (FG) has been shown to be susceptible to nucleophilic substitution to form sulfhydryl [45], amino [46–49], dichlorocarbene [50], or urea [51] groups for further functionalization (Fig. 1b). Regarding the chemical structure of FG, it has been considered as chemically inert material as an analogue to polytetrafluoroethylene (Teflon®) [52]. However, after Zbořil group reported reductive defluorination of FG [53], a number of methods have been developed for modification of FGs [54].

**Scheme 2:**
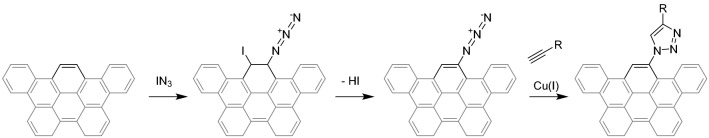
Modification of graphitic carbon with azides and coupling to terminal alkynes

Another example is the Cu^I^-catalyzed azide-alkyne cycloaddition (CuAAC) “click” reaction. The click reaction usually uses mild reaction condition and gives high conversion yield. Azide-modification of graphitic carbon surface can be simply carried out by either immersing them in acetonitrile solution of IN_3_ or exposing them to IN_3_ gas [55–57]. Further modification of carbon surface then can be carried out with CuAAC reaction (Scheme 2).

Metal-organic frameworks (MOFs) are emerging platforms for immobilization of molecular catalysts. Development of synthetic strategies of metal-organic frameworks (MOFs) provides rational design of molecular catalysts embedded in them. Two major constitutional components of MOF are metal ions/clusters and linking ligands, providing strategies to realize MOF catalysis based on (i) metal ions/clusters as catalytic sites or (ii) modification of organic ligands with molecular catalysts, respectively [58].

### Non-covalent immobilization

Non-covalent immobilization of molecular catalysts allows a facile mix-and-go strategy for catalyst preparation [59, 60]. A figure of merit for non-covalent interaction over covalent bonding is that due to the rather weak electronic interplay, the physical/chemical properties of both anchoring molecules and supporting matrices can be maintained. A number of non-covalent functionalization strategies, using π-π interaction, hydrogen bonding, electrostatic interaction, physical encapsulation, and mechanical interlocking, etc., have been reported so far.

**Fig. 2:**
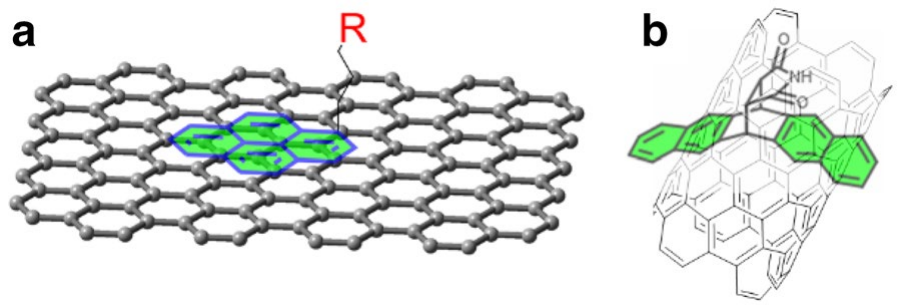
Schematic representation of a pyrenyl moiety sitting on the basal plane of graphene (**a**) and a Diels-Alder adduct of pentacene tweezing a single-walled carbon nanotube (**b**) through π–π interaction

Extended aromatic moieties such as pyrenyl group has been widely explored for immobilization of molecules on graphitic carbons based on the strong π-π interaction between them (Fig. 2a) [61–65]. Especially, a Diels-Alder adduct of pentacene has optimal dimension with the curvature of single-wall carbon nanotubes (CNTs), thus forms tweezer-like binding (Fig. 2b) [66]. The size-specific interaction enabled separation of CNTs mixture in 1–2 nm diameter range. For graphene oxide (GO), additional hydrogen-bond with epoxides, alcohols, ethers, and carboxylic acids can be formed in addition to the π–π interaction. The strong interaction of aromatic moieties with graphitic carbons was further evidenced by microscopic techniques [67–71].

Electrostatic interaction can also play a role to immobilize molecular catalysts. Oxidized CNTs or graphenes can afford strong ionic conjugates. Liang et al. demonstrated functionalization of GO and reduced graphene oxide (rGO) with ammonium salts [72]. Electrostatic functionalization of carbon support can be further expanded to end-functional polymers. Kim group reported stable dispersion of rGO in organic solvents by incorporating amine-terminated polystyrene [73]. Hydrophobicity/hydrophilicity of a molecule-support hybrid system can be effectively tuned by this technique, thus opens wider range of solvent selection.

## Photocatalysis

**Scheme 3:**
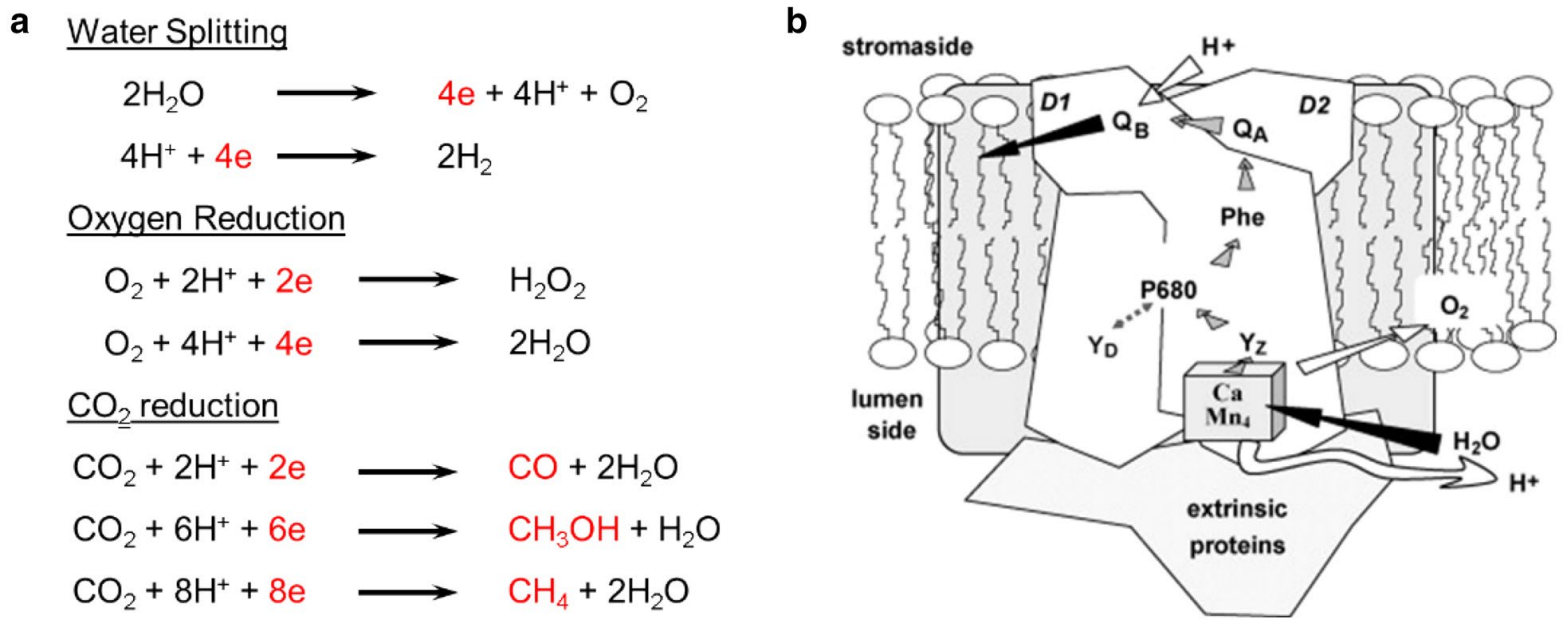
**a** Electron equivalents of various artificial photosynthetic processes. **b** Arrangement of subunits in PSII. Copyright permission from Elsevier [74]

Photocatalysis is a process where light energy is directly converted into chemical energy. It starts with absorption of photons by photosensitizers, followed by cascade charge/energy transfer to catalysts which convert substrates into products. Artificial photosynthesis schemes are multi-electronic processes, which implies multiple charges should be directed and accumulated at the catalytic center to run the photochemical cycles (Scheme 3a). It is a challenging subject, since photon absorption is generally a monoelectronic process, results in single charge production. In this context, it is required to accumulate electron/holes by multiple rounds of single electronic absorption before exciton decay, charge recombination, or back charge transfer. The best example is photosystem II (PSII) in chlorophyll, which is responsible for O_2_ evolution in natural photosynthesis. The active units in PSII are optimally positioned in a protein matrix so the charge transfer reactions are kinetically controlled to extract four electrons from a Mn_4_Ca cluster to run water oxidation (Scheme 3b) [74]. It is not straightforward to directly replicate the complex protein matrix of nature, however, efforts have been made to borrow the strategies in more simple chemical bonds or supramolecular chemistry [75–79].

### MOFs as immobilizing matrices

Many MOFs bearing aromatic ligands have broad UV–vis absorption assigned to metal-to-ligand charge transfer (MLCT), ligand-to-metal charge transfer (LMCT), or ligand-centered (LC) π–π* transition. This feature inspired researchers to use them as immobilizing matrices for molecular photosensitizers/catalyst for various photocatalytic reactions such as degradation [80–85] or transformation [86–89] of organic molecules, and organic photocatalysis.

**Fig. 3:**
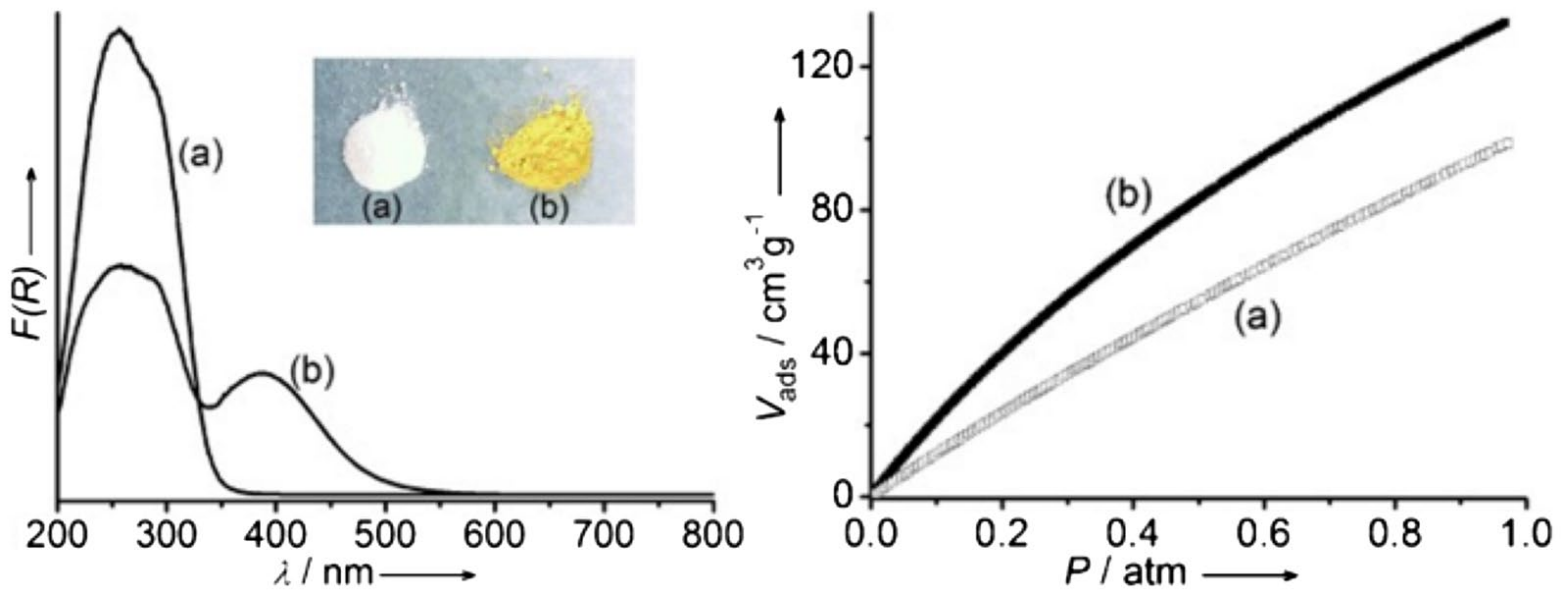
UV–vis absorption (left) and CO_2_ adsorption isotherms (1 atm, 273 K, right) of **MIL-125(Ti)** and **NH**_**2**_**-MIL-125(Ti)**. The inset photo shows absorption colors. Copyright permission from Wiley-VCH [93]

Ni-based MOFs are reported as visible-light driven photocatalysts for CO_2_ reduction. Simple ligand modification for PCN-222 and PCN-601 resulted in major products of formate anion and CH_4_, respectively [90, 91]. The result implies the importance of ligand selection, which affects the charge separation/transfer kinetics and the reaction sphere morphology. Li group introduced amino groups into the bridging ligands, resulting in increased catalytic activities for CO_2_ reduction compared to the MOFs without amino groups [92, 93]. Systematic studies on Ti-based MOFs, namely MIL-125(Ti) for amine-free MOF and NH_2_-MIL-125(Ti) for amino-functionalized MOF, showed the amine moiety on the ligands enhances light absorption in the visible range and increases adsorption of CO_2_ (Fig. 3).

Photocatalytic activities can be further enhanced by incorporating photosensitizers and/or catalysts into the MOFs. The UiO MOFs have been extensively studied as immobilizing matrix for molecular catalysts due to their facile ligand tunability and high chemical/photochemical stabilities. The UiO family has a configuration where Zr_6_(OH)_4_O_4_^12+^ clusters are 12-connected by organic dicarboxylate linkers to form a MOF framework with fcc structure. Various organometallic complexes can replace the linking ligands by filling the well-defined cavities while maintaining the structure of parent UiO MOF.

**Fig. 4:**
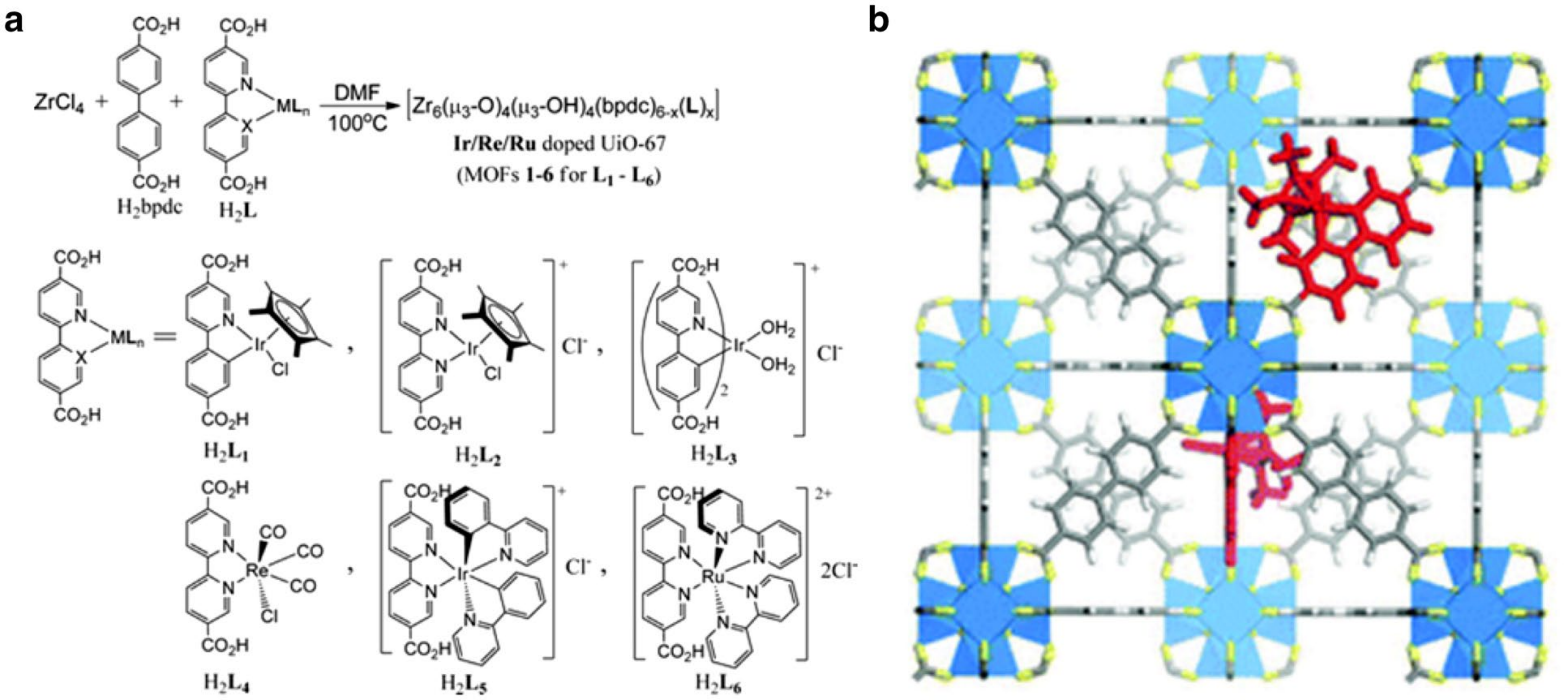
**a** Synthesis of organometallic catalysts doped **UiO-67**. **b** Structure of **L1**-doped **UiO-67**. The inset photo shows absorption colors. Copyright permission from ACS. [94]

Lin group reported a UiO-67 (Zr_6_O_4_(OH)_4_(bpdc)_6_, where bpdc is *p*-biphenyldicarboxylic acid) based MOF containing catalytically active Ir(III), Re(I), or Ru(II) complexes [94]. The MOFs were synthesized through mix-and-match strategy, owing to the matching ligand lengths between bpdc and metal-functionalized ligands (Fig. 4). MOFs **1**–**3** containing Cp*Ir(III)(dcppy)Cl (where Cp* is pentamethylcyclopentadienyl and dcppy is 2-phenylpyridine-5,4′-dicarboxylic acid) or Ir(III)(dcppy)_2_(H_2_O)_2_ were effective water oxidation catalyst with tyrnover frequencies up to 4.8 h^− 1^. The Re(I)(CO)_3_(dcbpy)Cl (H_2_**L**_**4**_, where dcbpy is 2,2′-bipyridyl-5,5′-dicarboxylic acid) derivatized MOF **4** served as an active catalyst for photocatalytic CO_2_ reduction with a total turnover number of 10.9, three times higher than that of the homogeneous complex H_2_**L**_**4**_.

**Fig. 5:**
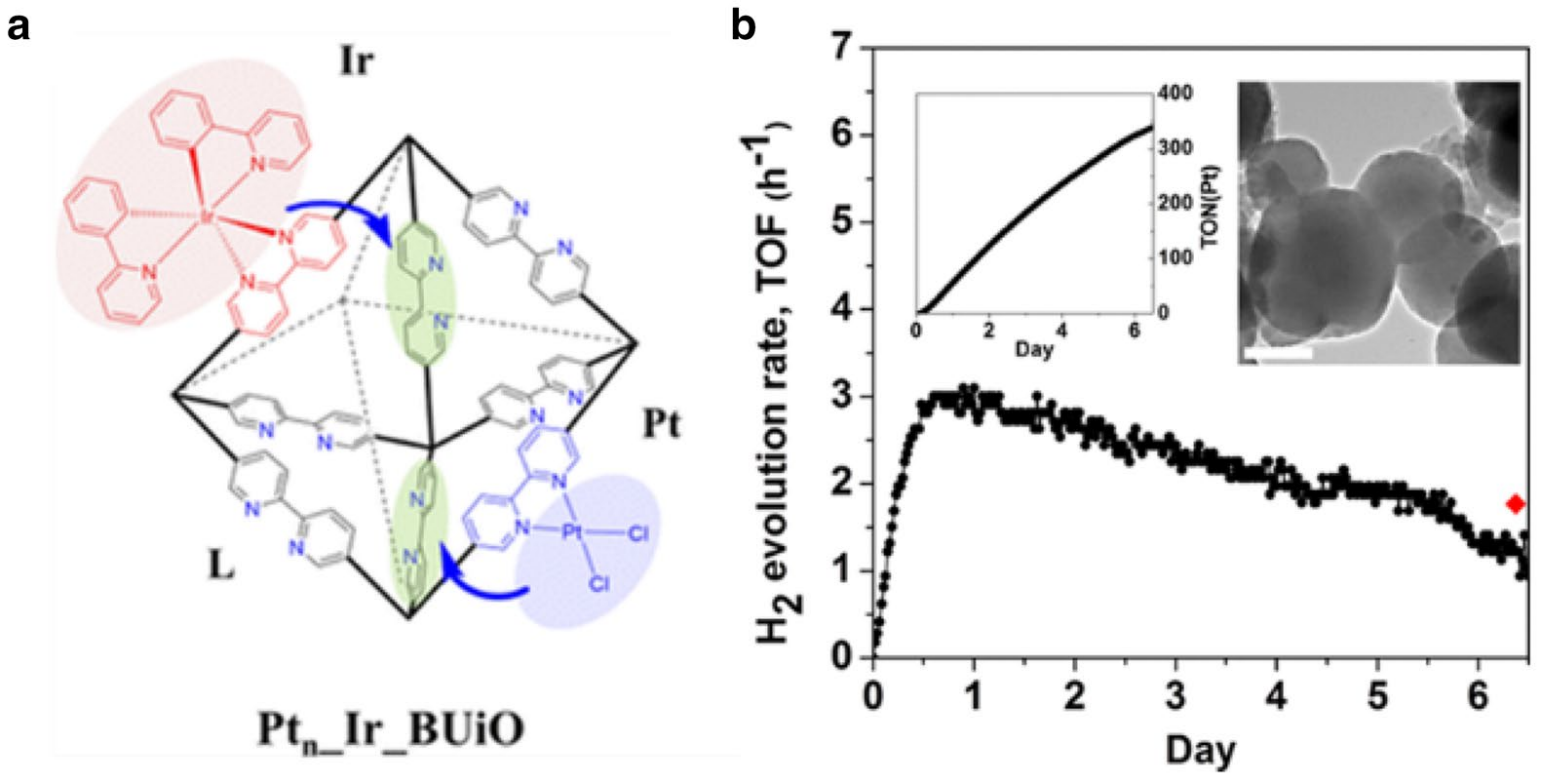
**a** Schematic operation principle of self-healing MOF (**Pt**_**n**_**_Ir_BUiO**). **b** H_2_ evolution rate curve of **Pt**_**0.1**_**_Ir_BUiO**. Inset: total amount of H2 evolved (left) and HR-TEM image after 6.5 days of photolysis. Scale bar = 200 nm. Copyright permission from ACS [95]

Kim et al. selected a bipyridine-embedded UiO-67 MOF, namely BUiO, as a self-healing platform for molecular Pt(II) catalysts and Ir(III) photosensitizers. A series of BUiO-based MOFs, namely, **Pt**_**n**_**_Ir_BUiO**, comprising 2,2′-bipyridine-5,5′-dicarboxylate (**L**) as a self-healing site, Pt(II)(**L**)Cl_2_ as a H_2_-evolving catalyst, and Ir(III)(ppy)_2_(**L**) as a photosensitizer were synthesized and tested for photocatalytic H_2_ production [95]. When the metal-diimine bonds were cleaved during photolysis, abundant free diamine ligands promoted re-coordination of the cleaved metal ions to recover the molecular catalytic activity (Fig. 5a). Accordingly, the catalysis with **Pt**_**0.1**_**_Ir_BUiO** prolonged more than 6.5 days without significant decrease in its activity, while the control MOF without a free diimine ligand lost molecular catalytic activity after 7.5 h by leaching of Pt and forming colloids (Fig. 5b).

### Dye-sensitized inorganic/organometallic hybrids

**Scheme 4:**
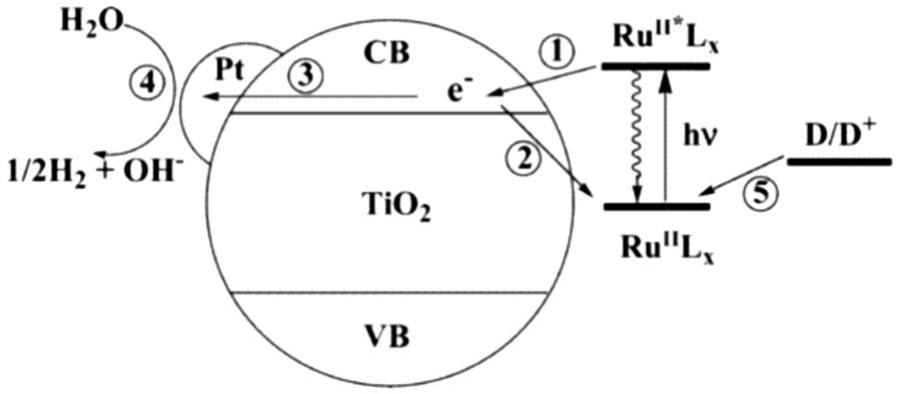
Visible-light-driven H_2_ production with Ru(II) dye-sensitized TiO_2_. Copyright permission from ACS [99]

In inorganic photocatalysis, metal oxides are typically studied due to their stability and facile synthesis. More importantly, multiple charges can be stored in a nanocrystal, which can be further utilized for multi-electronic photocatalytic cycles. Depending on the size of a nanocrystal, different maximum number of charges can be stored. Mayer and co-workers have shown a ZnO nanocrystal with 3.5 nm radius can take up to 120 electrons per particle [96]. Most of them work well with UV light and only a few visible-light-responsive photocatalytic systems are reported so far [97, 98]. Non-oxide nanocrystals such as CdS or CdSe have suitable bandgaps with strong visible-light absorption, which is suitable for artificial photosynthesis. However, the toxicity of Cd limits their usage in real life. As an analogue to dye-sensitized solar cells, hybridizing inorganic nanocrystals with organic/organometallic dyes offers visible-light driven photocatalysis. Scheme 4 illustrates electronic processes in a Ru(II)–TiO_2_/Pt system for photocatalytic H_2_ production: photoexcitation of a Ru(II) photosensitizer (**Ru**^**II**^**L**_**x**_), followed by (1) charge transfer from the photoexcited states of the Ru(II) dye (**Ru**^II^*L_**x**_) to TiO_2_ nanocrystal, (ii) back electron transfer from TiO_2_ conduction band to the dye, (iii) electron transfer to Pt catalyst, (iv) water reduction to produce H_2_, and (v) regeneration of the **Ru**^**II**^**L**_**x**_ ground state by an electron donor (**D**).

Kaneko and co-workers reported sensitization of TiO_2_/Pt nanoparticles with Ru(II) dyes for visible-light driven H_2_ evolution from water [100]. The study shows the importance of anchoring groups: Ru(II)(bpym)_3_^2+^ (where bpym is bipyrimidine) showed much higher H_2_ evolution rate (80.1 µl h^− 1^) than Ru(dcbpy)_3_^4−^ (17.8 µl h^− 1^)or Ru(bpy)_3_^2+^ (23.3 µl h^− 1^). Further investigation has been made on Ru(II)-sensitized TiO_2_/Pt to find structure-catalytic activity relationships for photocatalytic H_2_ production from water [99, 101–103].

Noble metal-free dye-sensitized systems have been also investigated. Yuan et al.. reported a noble metal-free H_2_ production system with Zn porphyrin sensitized MoS_2_/ZnO [104]. The 0.50 wt% ZnTCPP–MoS_2_/ZnO (where TCPP is 5,10,15,20-tetrakis(4-carboxyphenyl)-porphyrin) photocatalyst showed H_2_ evolution rate of 75 µmol h^− 1^ g^− 1^, which is comparable with the Ru(II)–TiO_2_/Pt photocatalysts. Li and co-workers reported a 5,10,15,20-tetraphenylporphyrin–Cu_2_O photocatalyst with H_2_ evolving rate of 1.3 mmol g^− 1^.

### Modification of carbon materials

**Scheme 5:**
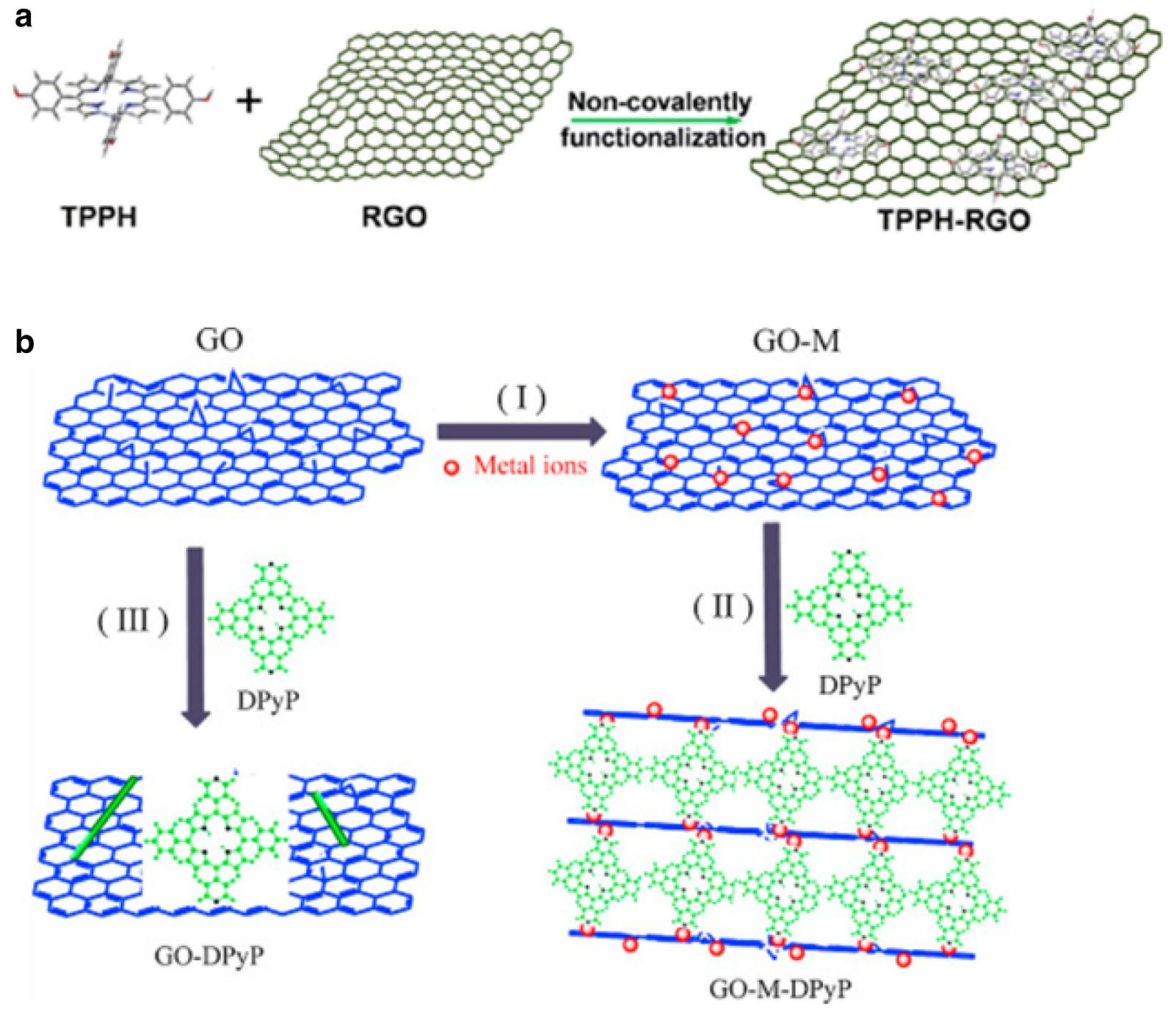
**a** Illustration of non-covalent incorporation of TPPH on the basal plane of rGO surfaces through Strong π-π interaction. **b** Formation of DPyP–Cr^3+^–GO (I and II) and DPyP–GO. Copyright permissions from ACS [105, 106]

Yang and co-workers reported a 5,10,15,20-tetrakis(4-(hydroxyl)phenyl) porphyrin (TPPH) non-covalently adsorbed on rGO with Pt nanoparticles [105]. Strong π–π interaction between TPPH and rGO offers stable photocatalysis (Scheme 5a). The TPPH–RGO/Pt catalyst showed an effective photocatalytic activity with a H_2_ production rate of 5.29 mmol g^− 1^. Li and co-workers reported Cr^3+^ implemented GOs bridged by 5,15-diphenyl-10,20-di(4-pyridyl)porphyrin (DPyP) [106]. Instead of forming π–π stacking between DPyP and GO, DPyP molecules are oriented to the out-of-plane direction of GO sheets by means of electrostatic and coordination interactions (I and II in Scheme 5b). The DPyP–Cr^3+^–GO showed visible-light-driven H_2_ production with activities of 928 µmol g^− 1^, while the control DPyP–GO (III in Scheme 5b) showed that of 686 µmol g^− 1^.

## Dye-sensitized photoelectrochemical (DS-PEC) cells

**Fig. 6:**
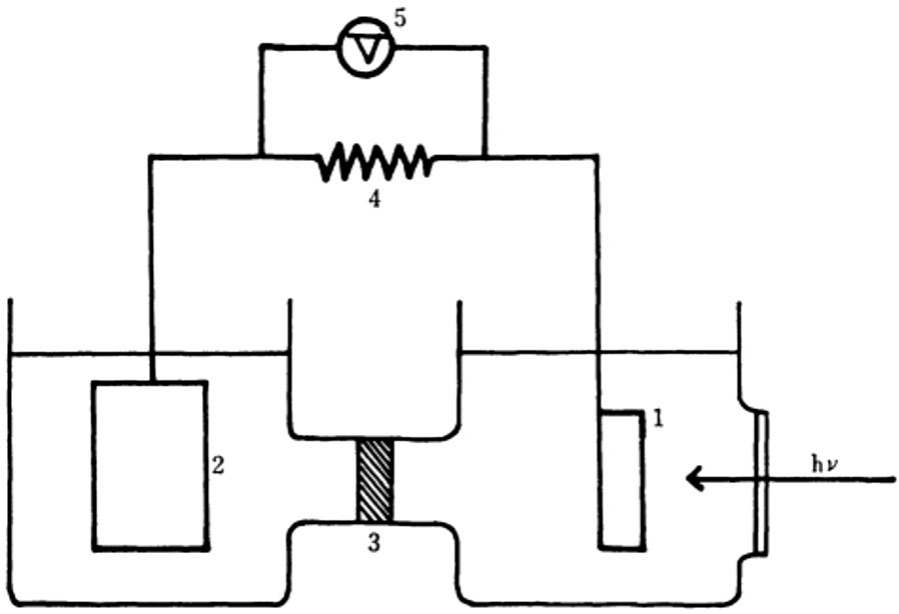
Illustration of a PEC cell reported by Honda and Fujishima: **1** is a TiO_2_ photoanode, **2** is a Pt counter electrode, **3** is a sintered glass diaphragm, **4** is an external load, and **5** is a voltameter. Copyright permission from The Chemical Society of Japan [107]

In the early 1970s, Honda and Fujishima reported a photoelectrochemical (PEC) water splitting cell comprising TiO_2_ photoanode and Pt counter electrode (Fig. 6) [107, 108]. By illumination of the photoanode (λ < 415 nm), O_2_ and H_2_ were generated at the photoanode and counter electrode, respectively. Since the pioneering works by Honda and Fujishima, significant progress has been made in pursuit of PEC water splitting systems using TiO_2_ photoanode or NiO photocathode. However, the large bandgaps of TiO_2_ and NiO limits the PEC cells operating with visible light. To tackle this issue, visible-light-absorbing semiconductors (VLA-SCs) have been studied, however, the catalytic activities of the VLA-SCs are rather lower than those of TiO_2_ or NiO [109, 110].

**Scheme 6:**
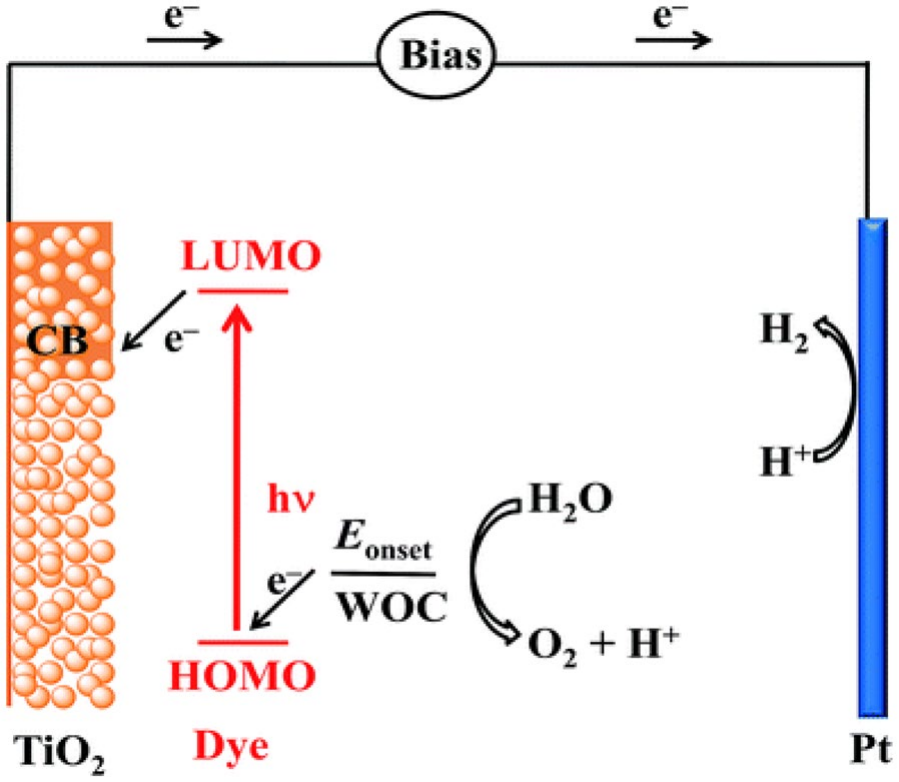
Schematic working principles of a water splitting DS-PEC with dye-sensitized DS-PEC cell. Copyright permission from RSC [112]

One promising strategy is sensitizing the large bandgap inorganic semiconductors with molecular photosensitizers. The concept of dye-sensitized solar cell (DSSC) was first proposed by O’Regan and Grätzel in 1991 [111]. Ru(II) complexes were adsorbed on the surface of TiO_2_ nanocrystals, which facilitates visible-light absorption by the Ru(II) dyes, followed by electrons from the photoexcited states injected into the TiO_2_ nanocrystals. The DSSC was first designed to generate electricity; same strategy can be applied to DS-PEC cells for catalytic reactions. Figure 5 depicts the working principle of a water splitting DS-PEC with dye-sensitized photoanode: (i) light absorption by dyes anchored to n-type semiconductor nanoparticles, (ii) injection of photoexcited electrons into the conduction band of semiconductor, (iii) extraction of electrons from water oxidation catalysts (WOCs), (iv) flow of electrons to counter electrode of Pt through outer circuit, and (v) catalysis on the surface of each electordes with supplied holes/electrons (Scheme 6). As an analogue to the dye-sensitized photoanode, dye-sensitized photocathode is also feasible, by incorporating dyes with p-type semiconductor nanoparticles.

**Fig. 7:**
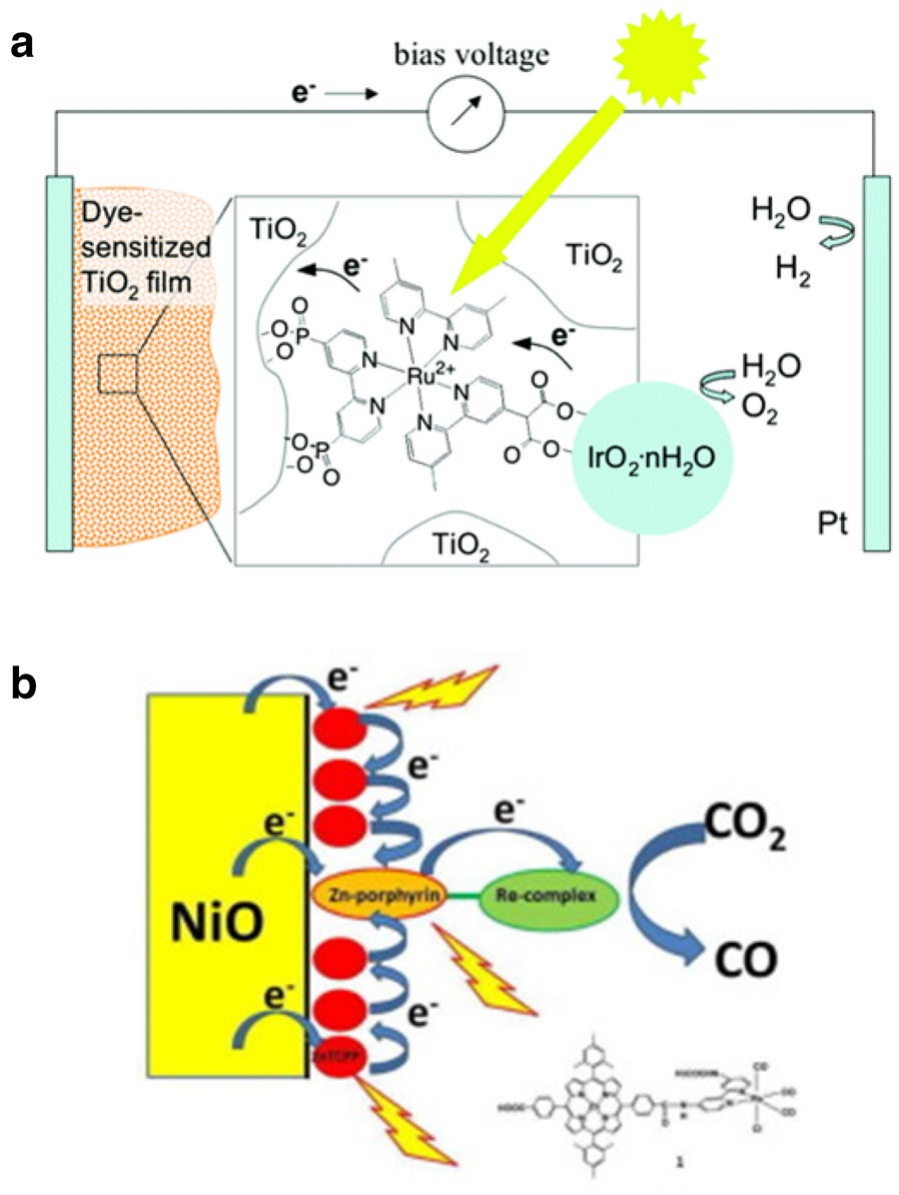
DS-PEC cells with **a** a dye-sensitized photoanode for water splitting and **b** a dye-sensitized photocathode for CO_2_ reduction. Copyright permissions from ACS and Elsevier [113, 128]

Mallouk and co-workers first reported a visible-light-driven water splitting DS-PEC cell [113]. Ru(II) dyes bridge TiO_2_ nanoparticles and IrO_2_·nH_2_O nanoparticles, directing cascade electron/hole transfer between them (Fig. 7a). Low internal quantum yield of 0.9% was reported due to: (i) slow electron transfer between IrO_2_·nH_2_O and oxidized Ru(II) dye and (ii) back electron transfer from TiO_2_ to the dye. To overcome the inefficient charge interplays, an electron mediator was incorporated to the photoanode, resulting in an increased internal quantum efficiency to 2.3%. Inspired by the works by Maooluk group, a number of dye-sensitized photoanodes for water splitting DS-PEC cells have been reported [114–127].

Reductive catalysis such as CO_2_ reduction or O_2_ reduction requires development of photocathodes with proper catalysts assembly. Inoue and co-workers reported a reduction of CO_2_ with dye-sensitized photocathode where a dinuclear Zn(II)–Re(I) complex is anchored to the surface of NiO nanoparticles (Fig. 7b) [128]. Although efficient hole injection efficiency from the dye to the NiO was observed, rather low Faradaic efficiency of 6.2% and turnover number of 10 were recorded due to decomposition of the sensitizing Zn(II) porphyrin part. With the same strategy, Ishitani group reported a dye-sensitized photocathode where Ru(II)–Re(I) dyads are anchored to NiO nanoparticles [129]. Enhanced Faradaic efficiency of 71% and turnover number of 32 were reported with the DS-PEC cell. Further improvement of Faradaic efficiency to 93% was achieved by replacing the NiO nanoparticles to CuGaO_2_ [130].

## Electrocatalysis

In a broader context, electrocatalytic fuel producing systems wired to external power sources, e.g. photovoltaic cells, is also categorized as artificial photosynthesis. Immobilization of molecular catalysts on electrodes requires few requirements for successful electrocatalysis: (i) charge transfer between electrode surface and immobilizing matrix shoud be assured, (ii) the immobilizing matrix should act as a charge transfer channel, and (iii) substrates and products can freely diffuse in the bulk of immobilizing matrix. By fulfilling these requirements, a number of electrocatalytic systems for water splitting, CO_2_ reduction, or O_2_ reduction have been reported.

**Fig. 8:**
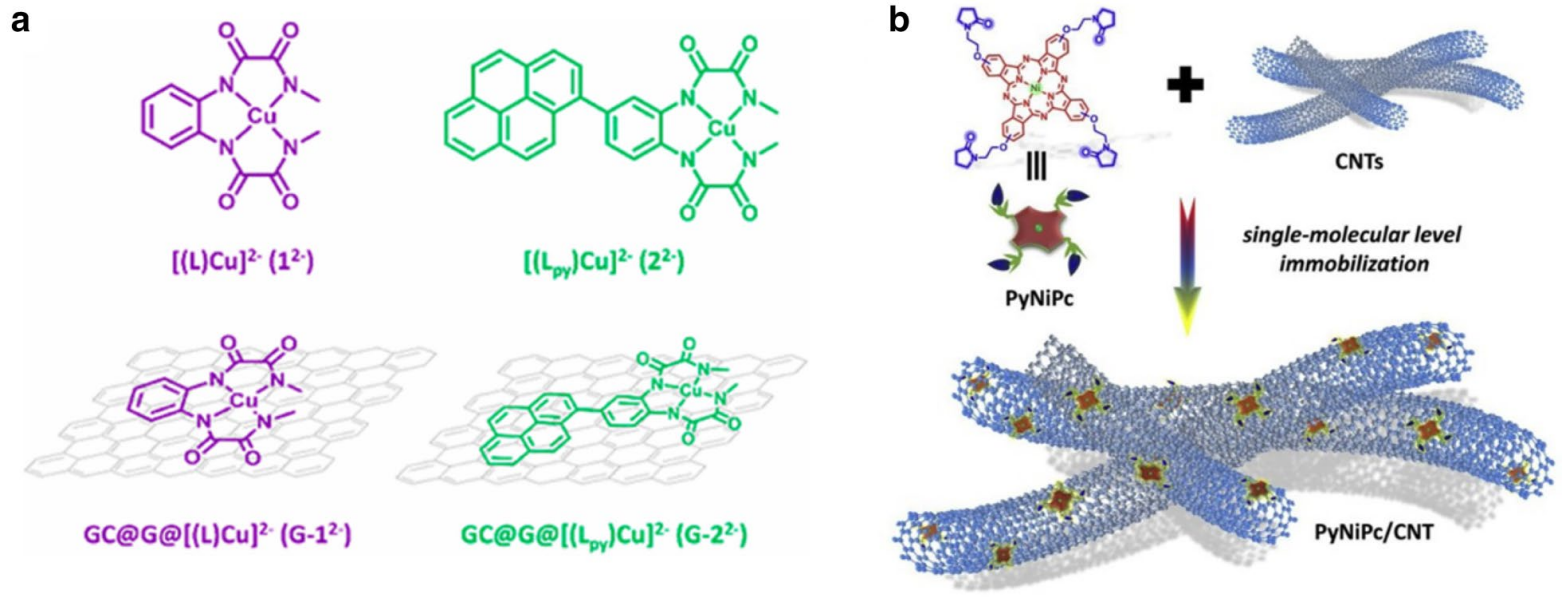
Adsorption of molecular catalysts on **a** the basal plane of graphene and **b** CNTs. Copyright permissions from Elsevier and ACS [133, 135]

A number of studies have been made in the development of molecular catalysts for homogeneous water splitting and they are well reviewed previously [21, 131, 132]. The huge library of water splitting catalysts provides facile way to directly immobilize them on the electrode surfaces for electrocatalytic systems. Garrido-Barros et al. demonstrated electrocatalytic water oxidation with Cu(II) complexes strongly bound on the basal plane of graphene sheets (Fig. 8a) [133]. Comparative study with the graphene-immobilized system (**G-2**^**2−**^) and a homogeneous system (**2**^**2−**^) as a control showed remarkable increase in catalytic activity: **G-2**^**2−**^ and **2**^**2−**^ showed maximum turnover frequencies of 540 s^− 1^ and 128 s^− 1^, respectively.

CO_2_ reduction in protic media inevitably competes with proton production, resulting in production of H_2_ as a byproduct. Interestingly, immobilization of electrocatalysts in solid matrices often enhances their selectivity to specific products by suppressing proton reduction. Wang and co-workers demonstrated highly selective CO_2_ electroreduction over H_2_ evolution with a Cu(I) complex adsorbed on graphene electrode [134]. It was proposed that the mesostructure of graphene favors diffusion of CO_2_ and limits mass transport of protons. More recently, Zhu and co-workers reported electrocatalytic CO_2_ reduction by immobilizing pyrrolidinonyl Ni(II) phthalocyanine (**PyNiPc**) on CNTs (Fig. 8b) [135]. The **PyNiPc**/CNT catalyst suppressed H2 evolution and selectively promoted CO_2_ reduction; a high CO/H_2_ value of 650 was recorded.

**Fig. 9:**
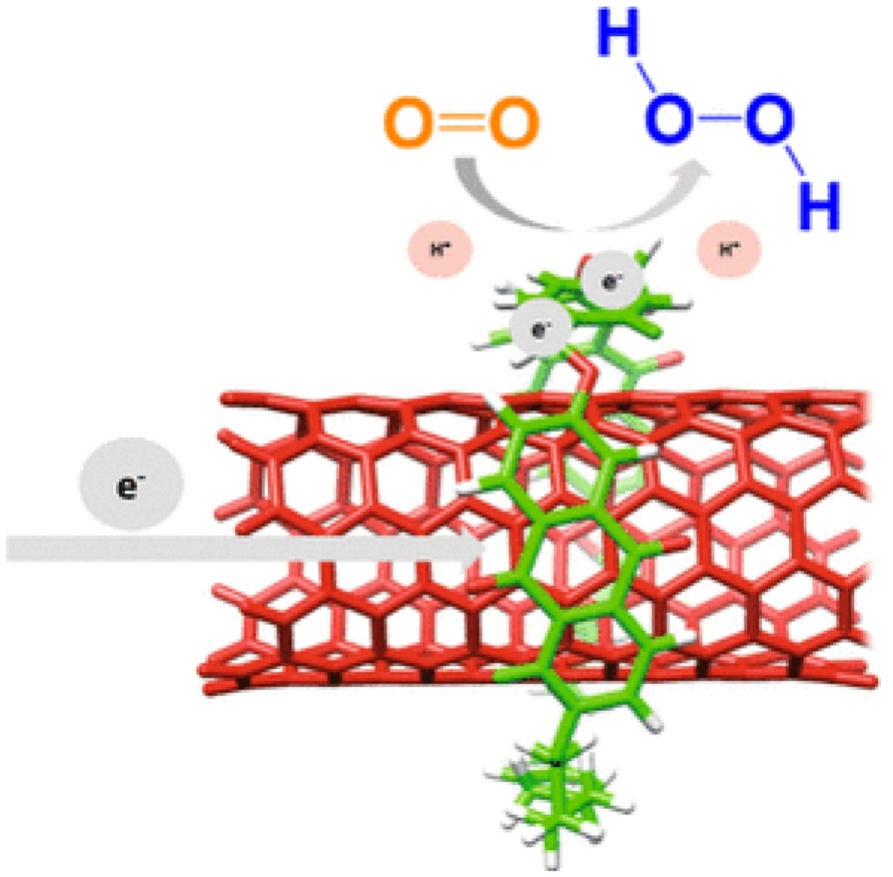
Electrocatalytic O_2_ reduction with a mechanically interlocked CNT with an anthraquinone macrocycle. Copyright permission from ACS [143]

Anthraquinone and its derivatives and its derivatives have been explored as molecular electrocatalysts for O_2_ reduction, since anthraquinone is low-cost, has suitable chemical functionality, and high selectivity for H_2_O_2_ [136–139]. Attempts to immobilize anthraquinones on electrodes comprise: (i) directly linking to glassy carbon electrodes through covalent bonds [140], (ii) incorporating in insoluble polymer matrix [141], (iii) and covalently linking to CNTs [142]. Wielend et al.. demonstrated a new approach of “mechanically interlocking” anthraquinone-based catalysts around CNTs [143] The rotaxane architecture prevents dissolution problems of physically adsorbed organic molecules upon electrochemical reduction, while retains the electrochemical properties of the pristine molecule (Fig. 9).

## Summary and outlook

The purpose of this review has been to understand the techniques to immobilize molecular catalysts in matrix-supports and incorporating them into artificial photosynthesis. Enhancement of catalytic activity and robustness was achieved with a simple chemistry using covalent bonds or non-covalent interactions. In addition, selectivity for specific product can be also enhanced by anchoring them in well-defined mesoporous structure. Synergetic effects were shown by hybridizing complementary molecular catalysts and solid supports.

The results provide a good blueprint for artificial photosynthesis; the researches so far are in a lab-scale and require next step forward for practical application of artificial photosynthesis in real life. Especially, proper device design is required to address the subjects listed below.

### Scalability

The global production of H_2_ reached 790 Mton in 2018 (https://www.iea.org) and the H_2_O_2_ market valued 2.49 billion USD in 2019, (https://www.grandviewresearch.com). H_2_ and H_2_O_2_ are exclusively produced by natural gas reforming and anthraquinone process, respectively. Both of the methods require high energy input; renewable method, such as artificial photosynthesis, is desired to substitute the production. In this regard, scaled artificial photosynthesis with proper device design is required to comply with the large numbers. Atmospheric CO_2_ concentration of 420 ppm is generally not high enough to directly run the catalytic reactions. The low CO_2_ solubility in water (ca. 1.5 g L^− 1^ at the standard condition) also limits the scalability of CO_2_ reduction. Techniques such as gas-phase electrolysis coupled with flow cells are under development to circumvent the issue.

### Products separation

Artificial photosynthesis comprises two half-reactions, resulting in at least two different products. In terms of monetary expenses, simple and cost-effective separation methods are required to separate a desired product from the admixture of reagents, substrates, and crude products. In water splitting, H_2_ and O_2_ are produced in a same phase of gas. Gas separation membranes are widely used to selectively collect H_2_ from the water splitting products. CO_2_ reduction is much more complicated due to a variety of possible products, *vide supra*. The gaseous mixture CO and H_2_ bubbles out from the reaction solution; it can be further separated or directly used as a syngas. The liquid phase separation requires rather high energy and complicated techniques.

Artificial photosynthesis is a promising, at the same time, a challenging subject. Multidisciplinary collaboration in chemistry, material science, biology, physics is required for successful realization of artificial photosynthesis.

## Data Availability

Not applicable.

## Abbreviations

CNT: Carbon nanotube
CuAAC: Cu^I^-catalyzed azide-alkyne cycloaddition
DS-PEC: Dye-sensitized photoelectrochemical
DSSC: Dye-sensitized solar cell
FG: Fluorinated graphene
GO: Graphene oxide
LC: Ligand-centered
LMCT: Ligand-to-metal charge transfer
MLCT: Metal-to-ligand charge transfer
MOF: Metal-organic framework
PEC: Photoelectrochemical
PSIIPSII: Photosystem II
rGO: Reduced graphene oxide
VLA-SC: Visible-light-absorbing semiconductor
WOC: Water oxidation catalyst
